# New Measurement Methods of Network Robustness and Response Ability via Microarray Data

**DOI:** 10.1371/journal.pone.0055230

**Published:** 2013-01-28

**Authors:** Chien-Ta Tu, Bor-Sen Chen

**Affiliations:** Laboratory of Control and Systems Biology, Department of Electrical Engineering, National Tsing Hua University, Hsinchu, Taiwan; National Institutes of Health, United States of America

## Abstract

“Robustness”, the network ability to maintain systematic performance in the face of intrinsic perturbations, and “response ability”, the network ability to respond to external stimuli or transduce them to downstream regulators, are two important complementary system characteristics that must be considered when discussing biological system performance. However, at present, these features cannot be measured directly for all network components in an experimental procedure. Therefore, we present two novel systematic measurement methods – Network Robustness Measurement (NRM) and Response Ability Measurement (RAM) – to estimate the network robustness and response ability of a gene regulatory network (GRN) or protein-protein interaction network (PPIN) based on the dynamic network model constructed by the corresponding microarray data. We demonstrate the efficiency of NRM and RAM in analyzing GRNs and PPINs, respectively, by considering aging- and cancer-related datasets. When applied to an aging-related GRN, our results indicate that such a network is more robust to intrinsic perturbations in the elderly than in the young, and is therefore less responsive to external stimuli. When applied to a PPIN of fibroblast and HeLa cells, we observe that the network of cancer cells possesses better robustness than that of normal cells. Moreover, the response ability of the PPIN calculated from the cancer cells is lower than that from healthy cells. Accordingly, we propose that generalized NRM and RAM methods represent effective tools for exploring and analyzing different systems-level dynamical properties via microarray data. Making use of such properties can facilitate prediction and application, providing useful information on clinical strategy, drug target selection, and design specifications of synthetic biology from a systems biology perspective.

## Introduction

Biological processes such as development, differentiation, tumorigenesis, and aging are increasingly being described in terms of temporal ordering of highly orchestrated transcriptional programs [1]. Although these processes can be analyzed using high-throughput gene expression microarray data at multiple time points, the computational methods available only identify which genes vary significantly, and how they vary across some or all of the time points measured [2], [3]. Despite the technological limitations, system-level properties such as robustness increasingly attracted serious scientific interest in systems biology. Robustness – the ability to maintain continued performance against intrinsic perturbations and uncertainty – has long been recognized as a key property of living systems and has been reviewed extensively elsewhere [4], [5]. Moreover, this fundamental and ubiquitously observed system-level phenomenon cannot be understood by focusing on the individual components, even though individual components of a system may or may not be robust themselves.

The term robustness is encountered widely in very different scientific fields, from engineering and control theory to dynamic systems [5] and biology [6], [7], [8]. It is important to note that robustness describes a relative property, not an absolute one, because no system can maintain stability in all functions when it encounters a perturbation. In other words, robustness is not immutable. If a system cannot alter itself, it cannot adapt to intrinsic perturbations or changes to its internal environment. Most robust biological systems possess a set of certain mechanisms to achieve this: in particular, both positive and negative feedback control are ubiquitous. Negative feedback, the main mechanism used to achieve a robust response to perturbations, promotes the restoration of an initial condition of a system. Positive feedback amplifies sensitivity to changes and perturbations, a necessary feature for a cell that needs to make decisions robustly [9], [10]. Therefore, feedback control is coupled with redundancy to enhance robustness. A signal transduction system is an additional network system that can be considered: it also plays an important role in responding to upstream signals or transducing environmental stimuli to downstream transcription factors in intracellular communication. From the signal energy point of view, the network response ability can be considered as system gain. Nevertheless, there is still no systematic method to measure the response ability of a network system at present.

In the last decade, with advances in experimental techniques, many researchers have utilized high-throughput data from DNA microarrays, yeast two-hybrid assays, co-immunoprecipitation, and ChIP-on-chip to study many kinds of bio-molecular networks. To this end, these kinds of data are often integrated to construct gene regulatory networks (GRNs) and protein-protein interaction networks (PPINs). These molecular networks have been demonstrated to have great potential for revealing the basic functions and essential mechanisms of various biological phenomena; this is accomplished by understanding biological systems on a system-wide, holistic level rather than in terms of their individual components [11].

Although the concept of robustness tradeoffs has been proposed [12], few studies have quantitatively and systematically calculated the robustness and response ability of a network system. The qualitative notion that a biological system that is highly tolerant to perturbations is robust is largely uncontested; yet, there is no generally accepted approach using microarray data that can analyze robustness quantitatively and systematically from a systems biology perspective. Moreover, in a GRN or PPIN, each component can transduce the effects of external stimuli downstream, but the response ability of the network from the global point of view cannot be experimentally measured for all network components. To solve this problem, we propose estimating the response ability of a GRN or PPIN via microarray data computations. In this study, we present a new measurement method, the Network Robustness Measurement (NRM), to estimate the relative robustness of a GRN or PPIN at different time stages of biological processes and under distinct biological conditions, checking their ability to tolerate intrinsic perturbations and uncertainties based on Lyapunov stability theory. Moreover, we also propose another novel measurement method, the Response Ability Measurement (RAM), to estimate the ability of a network system to respond to and then transduce external stimuli to downstream regulators.

In addition, we illustrate our approaches by applying them to two distinct network systems: a GRN and a PPIN. First, the NRM and RAM are applied to an aging-related GRN constructed via microarray data. We show that a GRN is more robust to intrinsic perturbations in the elderly than in the young, and as such possesses poor response ability to external stimuli. We then applied these methods to PPINs of fibroblast and HeLa cells under oxidative stress conditions. We observe that the PPIN of cancer cells possesses better robustness than that of normal cells under oxidative stress. Moreover, the response ability of the PPIN calculated from the cancer cells is lower than that of the healthy cells. Since “robustness” and “response ability” are two complementary antagonistic characteristics of a network system in the context of system performance (i.e., a more robust system will be less responsive, and vice versa [13]), measurements of these systematic properties could provide verification of the system characteristics of biological networks constructed from microarray data. Measuring the properties of such trade-offs between the robustness and response ability of a network system may provide insight into systemic changes that occur under different biological conditions. These generalized NRM and RAM methods represent effective tools for exploring and analyzing different systems-level dynamical properties. Making use of such properties can facilitate not only predictions and applications in gene therapy and drug target selection from the perspective of systems biology, but also help calibrate design specifications of robust synthetic biological networks for practical applications [14], [15], [16]. Therefore, the proposed methods may have great potential for analyzing and designing the next generation of synthetic GRNs or PPINs.

## Materials and Methods

Since we provide test cases of measuring the robustness and response ability of a GRN and a PPIN in the Results section, here we will focus on how to construct dynamic system models for a GRN and a PPIN from microarray data and other databases. Then using these dynamic models, we will propose two methods to measure their robustness and response ability.

### Constructing a Dynamic Model for a Gene Regulatory Network (GRN)

It is assumed that the network of genes of interest consists of *Z* genes, and the time-series microarray data used for constructing the GRN contains *K* time points. In order to take into account the delayed effects of molecular trafficking and cellular signal transduction, the GRN can be represented by the following linear discrete-time dynamic system:
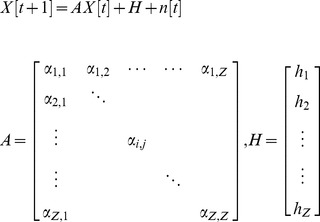
(1)where the state vector 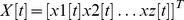 stands for the discrete-time mRNA expression levels of total *Z* genes at times 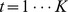. The system matrix *A* denotes the gene interactions in the gene network: i.e., 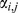 denotes the interaction from gene *j* to gene *i* when 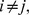 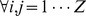; the constant vector *H* represents the basal level: i.e., *h_i_* denotes the basal level of the *ith* gene. *n*[*t*] denotes the model residue and measurement noise. If the GRN is nonlinear, then the linear gene network in (1) can be considered as the linearized system at the operation point of interest and *H* will be related to the location of the operation point of linearization: e.g., for a nonlinear gene network 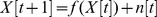 for some nonlinear interaction 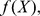 the linearized gene network around equilibrium point of interest 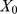 can be represented as 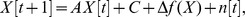 where 
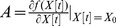
 and 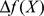 denotes the high order non-linear term. In this case, 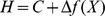 represents the basal level of the linearized system at this equilibrium point of interest. A case of a more general, nonlinear GRN will be discussed later.

*Remark 1:*

The network parameters of system matrix *A* and basal level *H* of GRN in (1) are identified by microarray data via recursive least square estimation method [17] in Text S1.

In the following subsections, we would like to measure robustness and response ability of a PPIN for a given biological phenomenon. Therefore, we need to construct the rough PPIN by data mining and then prune it using microarray data to obtain the refined PPIN for the phenomenon.

### Constructing the Rough Protein-protein Interaction Network (PPIN)

In order to obtain a PPIN for a biological phenomenon and investigate its network robustness and response ability, we first need to reconstruct the rough PPIN from microarray data and protein-protein interaction (PPI) information: in this case, our data was taken from STRING (Search Tool for the Retrieval of Interacting Genes/Proteins). STRING, a database of known and predicted protein interactions, comprises direct (physical) and indirect (functional) associations mined from four sources: genomic context, high-throughput experiments, conserved co-expression and previous knowledge. The database currently covers 5,214,234 proteins from 1,133 organisms. As a preliminary measure, we first perform a Bonferroni correction to select those genes with significantly differential expression (the corrected *P*-value = 0.01/*n*, where *n* = 25,802 denotes the number of genes) from microarray data for inclusion in the target pool. Bonferroni correction, one of the strictest and most conservative multiple testing corrections, adjusts the individual *P*-value for each gene to keep the overall error rate less than or equal to the user-specified *P*-cutoff value when performing a statistical test on a group of genes [18], [19]. Subsequently, this target pool and the PPI database (STRING) are integrated to indicate the possible PPIs between two selected candidates, and the rough PPIN can then be constructed based on the selected candidates in the protein pool and the potential PPIs among them.

### Pruning the Rough PPIN via a Dynamic Model

Modern large-scale studies of PPIs permit the development of protein interaction networks, but all large-scale experiments and databases to date have returned high rates of false-positives [20]. Previous evidence has clearly demonstrated that utilizing multiple functional databases not only allows for better identification of PPIs, but also leads to better prediction of the function of unknown proteins [21]. The rough PPIN constructed here comprehensively details the protein interactions possible under all kinds of biological situations and experimental conditions. As such, these interactions must be further narrowed down by microarray data so that they represent only the protein interactions appropriate for the conditions under consideration. Therefore, we use a dynamic PPI model and model order selection method, Akaike Information Criterion (AIC) [22], together to prune the rough PPIN using time series microarray data, deleting false positive PPIs in our PPIN. Here, a dynamic PPI model for a rough PPIN can be represented as follows:
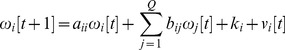
(2)where 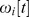 denotes the expression profile of the *i*th target protein 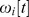 in the rough PPIN at time point 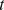; *v_i_*[*t*] denotes the model residue and measurement noise; 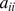 denotes the influence of the *i*th target protein on itself at the next time point; 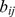 denotes the individual interactive ability of protein *j* in the rough PPIN with target protein 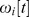 when 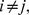
_
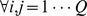
_ and the basal interaction *k_i_* represents unknown PPIs or other influences such as mRNA-protein interactions or protein synthesis. Therefore, the PPIN with dynamic protein interactions in (2) can be represented by



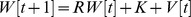
where
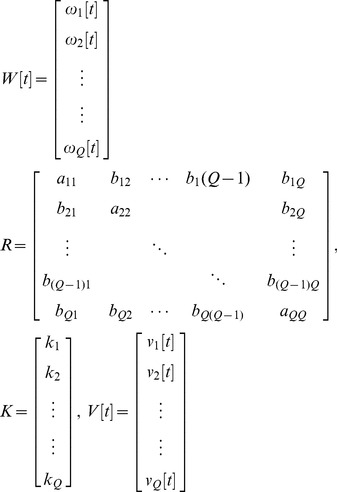
(3)where R denotes the interactive matrix of the PPIN, K denotes the basal levels of the PPIs, and V[t] denotes the modeling and measurement errors.

*Remark 2:*

The protein interaction model in (2) can be used to describe any target protein in the rough PPIN. Therefore, the whole PPIN can be described by the protein interaction model described in (3).

In equation (2), 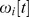 denotes the expression profile of the *i*th target protein at time point *t*, which is estimated from mRNA expression profiles through the following translational sigmoid function [23]:
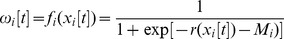
(4)where *r* denotes the transition rate of the sigmoid function, *M_i_* denotes the mean mRNA expression level of the corresponding protein *i*, and 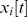 represents the corresponding mRNA expression profiles of the *i*-th target gene in the rough PPIN.

*Remark 3:*

The network parameters *R* and *K* of PPIN in (3) are also identified by the recursive least square estimation method [17]. Further a system order detection method, i.e. Akaike Information Criterion (AIC), is employed to detect the number *Q* of protein interactions in interactive matrix *R* in (3), i.e. use AIC to prune the false positive protein interactions in *R* (see Text S2).

### Network Robustness Measurement (NRM) of a Dynamic Network System

After constructing the dynamic models of the GRN in (1) and the PPIN in (3) from time series microarray data by the recursive least square estimation method as detailed above, we will measure their network robustness based on these dynamic models. The steady state 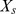 of the dynamic system in (1) is obtained as:
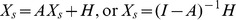
(5)


To simplify analysis of “robustness” of the steady state (phenotype) of the GRN, the origin of the dynamic system is shifted to the steady state 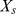
(i.e., 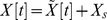), achieving the following shifted dynamic system by subtracting equation (1) from equation (5) [24].
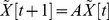
(6)


Therefore, the robustness of the network’s steady state becomes the robustness of the shifted network in (6) at the origin 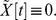 In the shifted dynamic system in (6), if the eigenvalues of *A* are all inside the unit circle 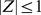 in the complex *Z* domain, then 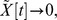 and 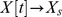, or approaches the steady state asymptotically: that is, the phenotype will still be maintained after a small perturbation 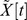. Since robustness of the dynamic system in (1) at the steady state 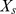 is equivalent to robustness of the dynamic system in (6) at the origin 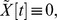
the shifted dynamic system in (6) will simplify the robustness analysis procedure of a network system [24], [25].

*Remark 4:*

The steady state 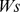 of the PPIN in (3) is obtained as:
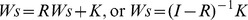



The origin of the dynamic PPIN is shifted to the steady state 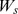 (i.e., 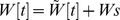), achieving the following shifted dynamic system [24].
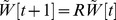
(7)


Therefore, the robustness of the PPIN phenotype becomes the robustness of the above shifted PPIN at the origin 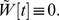 We then use this shifted PPIN in (7) to estimate its robustness and response ability in the same way as the GRN in (6) by following the same network robustness measurement (NRM) and response ability measurement (RAM) methods. Bearing this in mind, we only discuss the NRM and RAM of the GRN in (6). The measurements of NRM and RAM of a PPIN follow the same procedure.

The robustness of the shifted network in (6) is a measure of its ability to tolerate intrinsic molecular-level perturbation [13]. If the linear network system suffers from these kinds of perturbations, whether due to process noise, thermal fluctuations or genetic mutations, the interactive matrix *A* is perturbed as 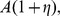 where 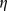 denotes the ratio of intrinsic perturbation, and the corresponding additional system perturbation 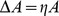 [13]. Then the network system with intrinsic perturbation considerations factored in can be represented by:
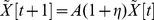
(8)


A higher 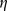 value means more intrinsic perturbations, and the larger the 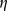 value that a network can tolerate, the greater robustness the network possesses. Since state-dependent intrinsic perturbations can influence the stability of a network system, we must further discuss the robustness of the perturbative network system in (8). According to quadratic stability theory [25], [26], if the Lyapunov equation of energy function for the perturbative network system in (8) is chosen as 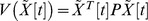 for a positive symmetric matrix 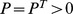, then the system in (8) is quadratically stable if 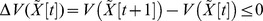: that is, if the energy of the GRN is not increased by intrinsic perturbations. Based on this idea, we are able to obtain the following robust stability principle for a GRN of interest that is subject to intrinsic perturbations.

*Proposition 1:* Suppose a network system suffers from intrinsic perturbations as described in (8). The perturbative network is robustly stable if the following inequality has a positive definite solution 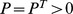:
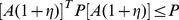
(9)


*Proof:* See Text S3.

*Remark 5:*

- If a network system is free of intrinsic perturbations as in (6) (i.e., 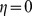), the stable condition is reduced to the following matrix inequality, 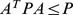
_,_ which has a positive definite solution 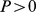 [13]. In order to guarantee the stability of a perturbation-free network system, eigenvalues of the system interaction matrix *A* should be inside the unit circle 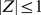 in the Z complex domain: i.e., 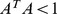. From (9), it can be seen that when 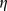 is increased gradually, eigenvalues of system interactive matrix *A* of the perturbation system in (8) should be nearer the origin of the complex domain *Z* than those from the perturbation-free system in (6) in order to tolerate more intrinsic perturbation 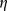. Some eigenvalues of system interactive matrix *A* near the unit circle are more easily perturbed outside the unit circle by intrinsic fluctuations, making the perturbative network system unstable. Therefore, the distance between eigenvalue locations of *A* to the unit circle represents a measurement of robustness for a linear network system.
- Based on the above analysis, the robustness 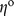 of our network system is the maximum perturbation tolerance allowed by the network, and it can be measured by solving the following constrained optimization:
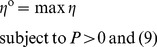
(10)i.e., the maximum perturbation ratio 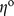 tolerable by the network system while maintaining stability. The constrained optimization problem in (10) can be solved by increasing 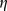 until no positive solution 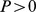 exists in (9): i.e., to the highest possible 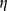 without violating the robust stability in (9). A positive definite solution 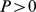 in (9) can be easily obtained by using the Linear Matrix Inequality (LMI) Toolbox of Matlab.

We can solve the above constrained optimization problem to measure the robustness of the network system simply by increasing 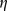 until no positive definite solution 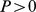 can be found in (9), and then the maximal perturbation 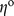 tolerated by the network system represents the robustness under intrinsic perturbations. Hence, we are able to obtain the relative robustnesses of network systems we would like to investigate under many different biological circumstances. When comparing 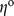 for each network, the higher the value of 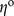, the greater the robustness estimate for its respective network.

### Response Ability Measurement (RAM) of a Dynamic Network System

In this subsection, we turn to how to estimate the response ability of a network system to external stimuli after its robustness has been calculated. Assuming a network system responds to external stimuli 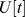, which might include upstream regulatory signals and external signals from factors such as carcinogens, oxidative stress and ambient pro-inflammatory molecules, the dynamic network (6) is then modified as follows [27].
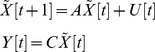
(11)where 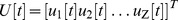
represents external stimuli and 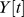 denotes the output signal responses of the network system of interest. For example, if the output signal response of all genes within the network system is analyzed, then 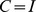, an identity matrix. If only the response of the last gene to 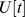 is analyzed, the matrix will be 
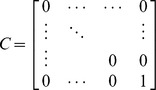
: i.e., all elements of *C* are zero except for the last element.

In a network system, each component may amplify or attenuate the effect of external stimuli when communicating downstream. The effect of input signals 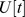 on output signals 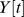 is less than or equal to a positive value 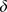, if the following inequality [25], [26]:
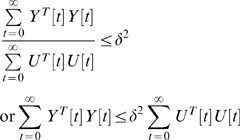
(12)holds for all possible bounded input signals 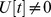 and 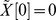. The physical meaning attributable to (12) is that the effect of all possible external stimuli 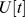 on 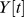 is less than or equal to 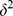 from the energy point of view: i.e., 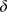 denotes the upper bound of the effect of 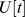 on 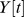. If 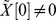, then inequality (12) should be modified as the following [25], [26]:
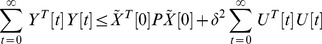
(13)to account for the effect of 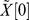 on 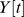. 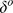, the smallest upper bound of 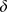 in (12), is called the “network response ability” of the system to all bounded stimulus signals 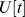. From 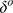, we obtain a more systematic insight into the ability of a network to respond to external stimuli, which is useful when considering signal transduction or intracellular communication. Via this method, we can measure and compare network response abilities 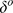 of various network systems under different biological conditions and states.

*Proposition 2:* The response of the network system in (11) has an upper bound 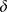 in (12), if there exists a positive definite 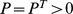 solution to the following linear matrix inequality (LMI) [28]:
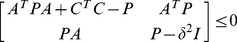
(14)i.e., if the above LMI holds for some 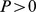, then the effect of 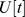 on 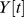 must be less than or equal to 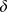: i.e., (12) or (13) hold for the network system in (11).

*Proof:* See Text S4.

*Remark 6:*

- The response ability 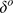
of a network system to external stimuli can be obtained by minimizing its upper bound 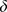, achieved by solving the following constrained optimization problem:

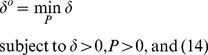
(15)This constrained optimization can be solved by decreasing the upper bound 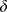 in (12) until no 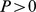 exists in (14) using Matlab LMI toolbox [27].

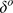
, the smallest upper bound of 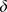 in (12), is called the network response ability of a network system to external stimuli. If 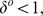 it means external stimuli are attenuated (buffered) by the network system; if 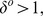 external stimuli are amplified.
- Since the output/input signal energy ratio (i.e., the response) in (12) or (13) is considered for all possible external stimuli, the measure of network response ability in (14) and (15) is dependent more on system matrix A than on the external stimuli. This is analogous to the way a lowpass filter is dependent more upon the characteristics of the filter than on noise. Therefore, it is possible to measure the network response ability of a network system from its system characteristics without knowledge of what the external stimuli are, with the caveat that these signals must be bounded. The system matrix A can be estimated from time-series microarray data as in the previous subsections. Therefore, employing the Matlab LMI toolbox, we can use (15) to measure network response abilities using microarray data under different specific experimental conditions.

### Extension of Network Robustness and Response ability Measurements to Nonlinear Network Systems

Suppose the GRN or PPIN could be constructed by microarray data for the following nonlinear network system:
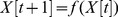
(16)


Suppose we are interested in the phenomenon around the equilibrium point 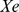
– i.e., 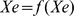 – and let 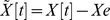. Then, we get the shifted nonlinear network system as:
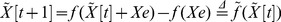
(17)


In this system, 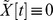 is the equilibrium point of interest. Suppose the nonlinear network in (17) could be globally linearized as described in [27] for all 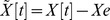 as:
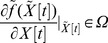
(18)where 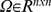 denotes the system parameter set of these globally linearized systems. Suppose 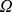 could be described by a list of its vertices via the following convex hull:



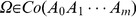
(19)By the global linearization technique described in [27], all the globally linearized systems in 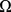 of the nonlinear system in (16) or (17) could be interpolated by the *m* linear system 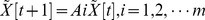 at the vertices of convex hull of the polytope:
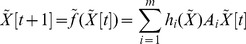
(20)where the interpolation functions 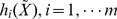 have the following properties:
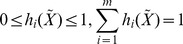
(21)i.e., the trajectory of the nonlinear system in (16) or (17) could be interpolated by the trajectories of m linear systems in (20) via the nonlinear interpolation functions 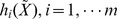


Suppose the nonlinear network system suffers from the following intrinsic perturbations:
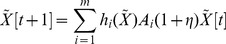
(22)


Based on the robust stability principle, we deduce the following.

*Proposition 3*: Suppose the nonlinear network system suffers from intrinsic perturbations as in (22). The perturbative nonlinear network system is robustly stable if the following LMIs have a positive definition 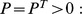


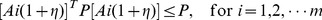
(23)


*Proof:* See Text S5.

The physical meaning of the LMIs in (23) is that the perturbation 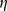 is tolerable within all linearized network systems. Therefore, based on the global linearization method, the robustness 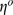 of the nonlinear network system in (16) can be measured by solving the following constrained optimization:
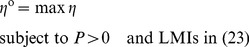
(24)i.e., the maximum perturbation 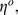 which is tolerable by all linearized network systems, is a global extension of (15) from a linear network system to a nonlinear network system.

Similarly, if the shifted nonlinear network system with output 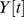 suffers from external stimuli as in:
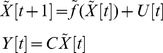
(25)then, based on the global linearization technique detailed in [27], the shifted nonlinear network system of (25) with output 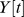 can be represented by the following interpolated system:



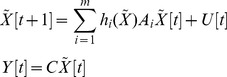
(26)Then we get the following result:

*Proposition 4*: The network response of the nonlinear network system in (26) has an upper bound 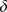 in (12), if there exists a positive definite solution 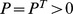 to the following LMIs:
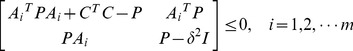
(27)i.e., if the above LMIs hold for some 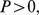 then the effect of 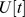 on 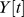 must be less than or equal to 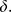 That is, (12) or (13) holds for the nonlinear network system (25) or (26).

*Proof:* See Text S6.

Therefore, based on the global linearization method, the network response ability 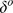 of the nonlinear network system in (25) or (26) can be measured by solving the following constrained optimization:
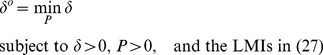
(28)


*Remark 7:*

If the nonlinear network system is constructed by the system identification method from microarray or other experimental data, then the network robustness 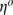 and response ability 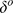 can be measured by (24) and (28), respectively. However, to approximate the nonlinear network system via interpolation, these systems must undergo the global linearization in (18) and (19) to find the vertices of the convex hull of the polytope in order to interpolate this linearized system at the vertices (as in the system in (26)). This is generally a very complex task. If we only want to measure the network robustness and response ability of a network system near an equilibrium point (e.g. phenotype), then the measurement methods for network robustness and response ability by (10) and (15) based on a linearized system at its equilibrium point are sufficient. In the following section, we give several practical examples based on linear network systems for measuring the network robustness and response ability of a GRN and PPIN constructed by time-series microarray data.

## Results/Discussion

**Figure 1:**
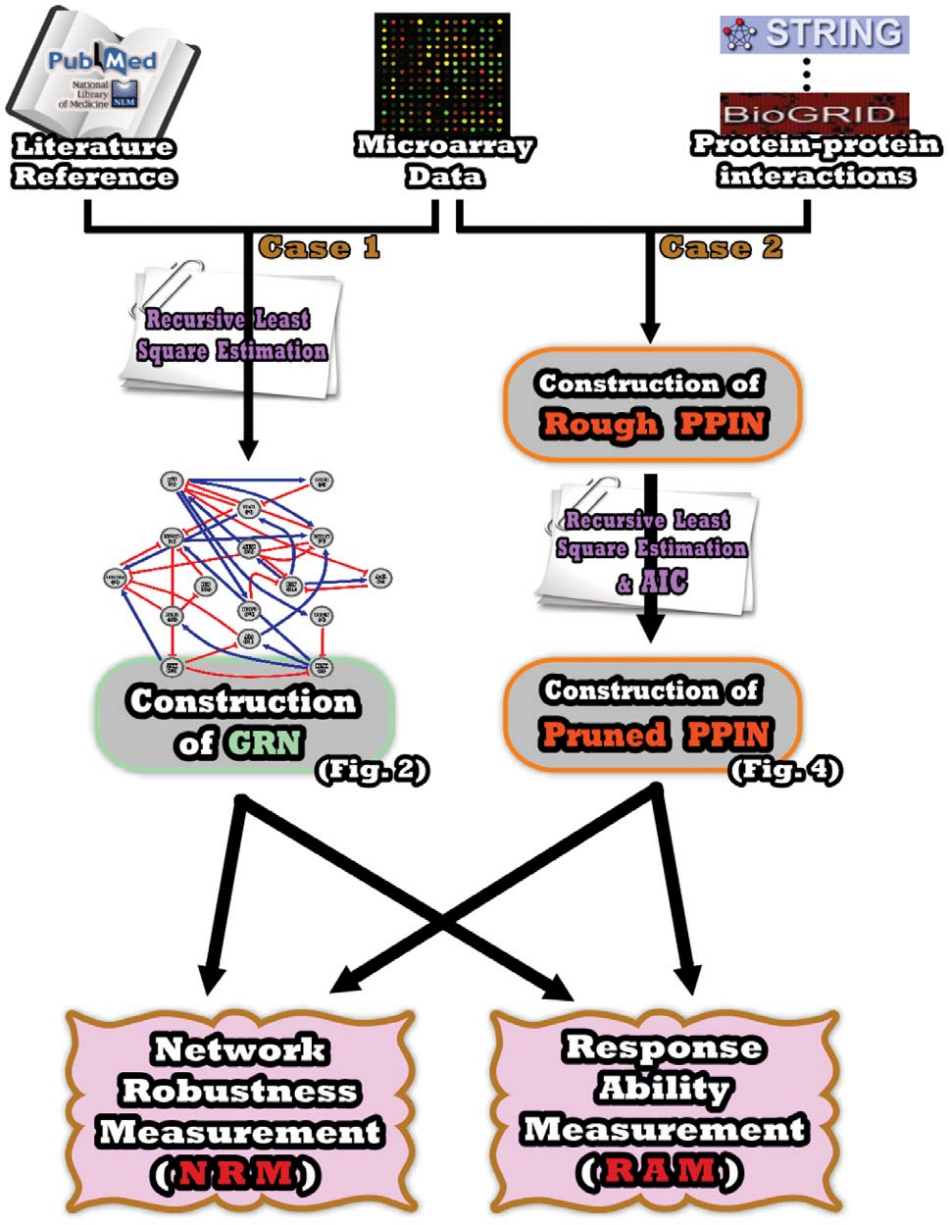
Flowchart of the proposed methods to estimate network robustness and response ability. This flowchart delineates the process used to construct the gene regulatory network (GRN) and protein-protein interaction network (PPIN), and the subsequent estimation of network robustness and response ability by the NRM and RAM methods, respectively. The flow chart on the left represents the Case 1 study analysis of the GRN; the flow chart on the right represents the Case 2 study, where methods are applied to a PPIN. [AIC-Akaike Information Criterion].

In this paper, we have presented two novel computational methods, Network Robustness Measurement (NRM) and Response Ability Measurement (RAM), which estimate the robustness and response ability of network systems using time-series microarray data. Briefly, the basic methodologies consist of integrating time-series microarray data with either experimental literature or protein-protein interaction information to construct the dynamic system of a gene regulatory network (GRN) or protein-protein interaction network (PPIN). Both of these systems correspond to real phenomena in living organisms and can be used to further evaluate network robustness and response ability (see Figure 1). Details have been described in the preceding Materials & Methods section. These methods are equally applicable to and useful for GRNs and PPINs. In order to demonstrate the potential applications of NRM and RAM for estimating system characteristics exhibited under different biological conditions, we elaborate on the usage of the above methods in two separate cases and explain the analytic results within the following subsection.

### Case 1: Estimating the Robustness and Response Ability of a Gene Regulatory Network (GRN) at Different Stages of Life

Aging is an extremely complex and system-level process, and it has attracted much attention in medical research, especially since chronic diseases are quite prevalent in elderly populations. These illnesses may be the result of both gene mutations that lead to intrinsic perturbations and environmental changes that stimulate signaling in the body. In addition, aging may result in reduced responses to environmental stimuli such as carcinogens, oxidative stress, and pro-inflammatory molecules. Therefore, measuring robustness to intrinsic perturbations and response ability to external stimuli of an aging-related GRN could provide key insights into the system-level mechanisms involved in aging.

given GRN, the more robust the GRN is.

#### Construction of a gene regulatory network associated with aging-related pathophysiological phenotypes

**Figure 2:**
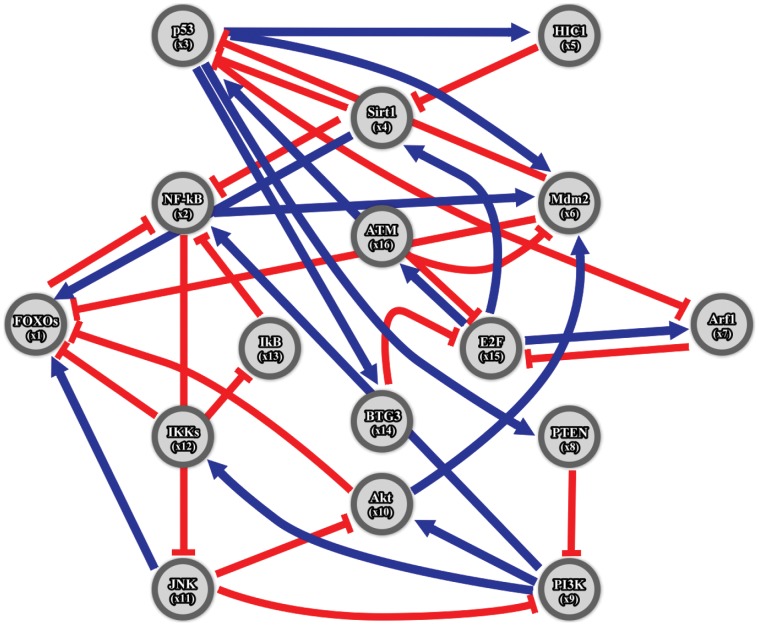
Multiple regulatory loops of a GRN associated with aging-related pathophysiological phenotypes. This network includes the following sixteen genes: FOXOs, NF-κB, p53, SIRT1, HIC1, Mdm2, Arf1, PTEN, PI3K, Akt, JNK, IKKs, IκB, BTG3, E2F1, and ATM. Blue arrows indicate activation; blunt red arrows indicate suppression.

Combing data from several references in the literature, we construct a GRN with multiple regulatory loops that are highly associated with aging-related pathophysiological phenotypes, consisting of the following sixteen genes: FOXOs, NF-κB, p53, SIRT1, HIC1, Mdm2, Arf1, PTEN, PI3K, Akt, JNK, IKKs, IκB, BTG3, E2F, and ATM (Figure 2). All genes of interest are critical for regulating cellular functions related to longevity, including detoxification of reactive oxygen species (ROS), cell cycle arrest, repair of damaged DNA, apoptosis, and pro-inflammation, as age increases [29], [30]. The GRN can be represented by the linear discrete-time dynamic system shown in Table 1.

**Table 1:** Ordinary differential equations of gene regulatory networks in Figure 2 for sixteen genes associated with aging-related pathophysiological phenotypes.

Gene		Dynamic Equations of Sixteen Genes of Interest
x_1_	FOXOs	*x*_1_[*t*+1] = *α*_1,1_ *x*_1_[*t*] + *α*_1,4_ *x*_4_[*t*] + *α*_1,6_ *x*_6_[*t*] + *α*_1,10_ *x*_10_[*t*] + *α*_1,11_ *x*_11_[*t*] + *α*_1,12_ *x*_12_[*t*] + *h*_1_
x_2_	NF-κB	*x*_2_[*t*+1] = *α*_2,1_ *x*_1_[*t*] + *α*_2,2_ *x*_2_[*t*] + *α*_2,4_ *x*_4_[*t*] + *α*_2,9_ *x*_9_[*t*] + *α*_2,13_ *x*_13_[*t*] + *h*_2_
x_3_	p53	*x*_3_[*t*+1] = *α*_3,3_ *x*_3_[*t*] + *α*_3,4_ *x*_4_[*t*] + *α*_3,6_ *x*_6_[*t*] + *α*_3,16_ *x*_16_[*t*] + *h*_3_
x_4_	Sirt1	*x*_4_[*t*+1] = *α*_4,4_ *x*_4_[*t*] + *α*_4,5_ *x*_5_[*t*] + *α*_4,15_ *x*_15_[*t*] + *h*_4_
x_5_	HIC1	*x*_5_[*t*+1] = *α*_5,3_ *x*_3_[*t*] + *α*_5,5_ *x*_5_[*t*] + *h*_5_
x_6_	Mdm2	*x*_6_[*t*+1] = *α*_6,2_ *x*_2_[*t*] + *α*_6,3_ *x*_3_[*t*] + *α*_6,6_ *x*_6_[*t*] + *α*_6,7_ *x*_7_[*t*] + *α*_6,10_ *x*_10_[*t*] + *α*_6,16_ *x*_16_[*t*] + *h*_6_
x_7_	Arf1	*x*_7_[*t*+1] = *α*_7,3_ *x*_3_[*t*] + *α*_7,7_ *x*_7_[*t*] + *α*_7,15_ *x*_15_[*t*] + *h*_7_
x_8_	PTEN	*x*_8_[*t*+1] = *α*_8,3_ *x*_3_[*t*] + *α*_8,8_ *x*_8_[*t*] + *h*_8_
x_9_	PI3K	*x*_9_[*t*+1] = *α*_9,8_ *x*_8_[*t*] + *α*_9,9_ *x*_9_[*t*] + *α*_9,11_ *x*_11_[*t*] + *h*_9_
x_10_	Akt	*x*_10_[*t*+1] = *α*_10,9_ *x*_9_[*t*] + *α*_10,10_ *x*_10_[*t*] + *α*_10,11_ *x*_11_[*t*] + *h*_10_
x_11_	JNK	*x*_11_[*t*+1] = *α*_11,2_ *x*_2_[*t*] + *α*_11,11_ *x*_11_[*t*] + *h*_11_
x_12_	IKKs	*x*_12_[*t*+1] = *α*_12,9_ *x*_9_[*t*] + *α*_12,12_ *x*_12_[*t*] + *h*_12_
x_13_	IκB	*x*_13_[*t*+1] = *α*_13,12_ *x*_12_[*t*] + *α*_13,13_ *x*_13_[*t*] + *h*_13_
x_14_	BTG3	*x*_14_[*t*+1] = *α*_13,3_ *x*_3_[*t*] + *α*_14,14_ *x*_14_[*t*] + *h*_14_
x_15_	E2F	*x*_15_[*t*+1] = *α*_15,7_ *x*_7_[*t*] + *α*_15,14_ *x*_14_[*t*] + *α*_15,15_ *x*_15_[*t*] + *α*_15,16_ *x*_16_[*t*] + *h*_15_
x_16_	ATM	*x*_16_[*t*+1] = *α*_16,15_ *x*_15_[*t*] + *α*_16,16_ *x*_16_[*t*] + *h*_16_

In this case, we want to estimate the network robustness and response ability of this GRN, and also to investigate whether they might be altered with increasing age. Furthermore, those altered system characteristics associated with a high incidence of aging-related disease, such as cancer, are prime targets for discussion. The high-throughput microarray data assembled in [31], which details the age-related effects on gene expression in the thymus and spinal cord dissected from male C57BL/6 mice of ages 1, 6, 16, and 24 months, are used as our gene expression profiles. In order to make this data more useful for identifying the system parameters of our dynamic GRN, we first rescale the original gene expression time profiles. For young mice, those aged 17d and 19d are categorized as 3-week-old animals; 40d, 43d, and 44d as 6-week-old animals; 174d, 180d, and 186d as 25-week-old animals; and, 193d and 194d as 28-week-old animals. For aged mice, those aged 476d, 481d, and 485d are categorized as 68-week-old animals; 495d and 498d as 71-week-old animals; 714d and 719d as 102-week-old animals; and, 730d, 733d, and 743d as 105-week-old animals. (All animals had been placed on *ad libitum* diets.) All of the above expression level data are calculated in log2 scale, and then the Z-score method is used to normalize the gene array expression data as has been previously described [32].

**Figure 3:**
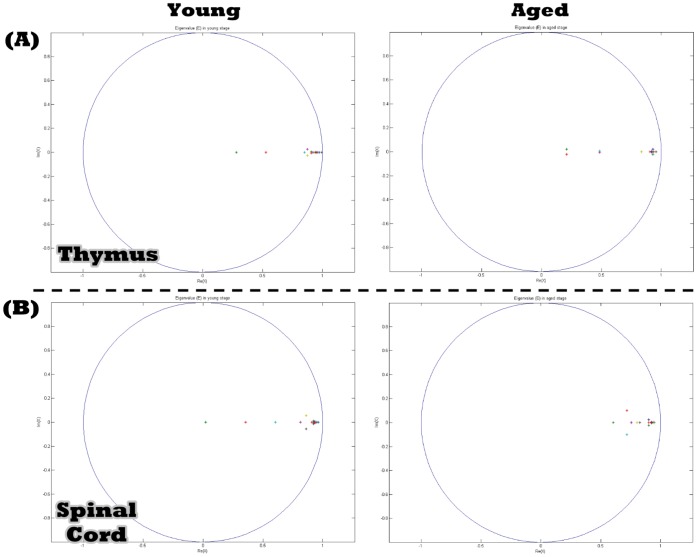
The locations of the sixteen eigenvalues of different tissues at the young and aged stages, respectively. Some eigenvalues of the interactive matrix *A* for the thymus (A) and spinal cord (B) are located together at similar regions near the unit circle |Z|  = 1.

We construct multiple regulatory loops of a GRN containing sixteen genes (Figure 2) associated with aging-related pathophysiological phenotypes. Using the recursive least square parameter estimation method [17] and the microarray data from the thymus and spinal cord [31], a dynamic model of the sixteen genes within the GRN in equation (1) is constructed (shown in Table 1) with estimated parameters at different stages of life (summarized in Table 2). With equation (1) and Tables 1–2, we calculate sixteen eigenvalues for interactive matrix *A* at the young and aged stages. The result (Figure 3) shows some eigenvalues of the interactive matrix *A* clustered together in similar regions near the point Z = 1, demonstrating that some modes of the same network will more readily approach some constant steady state than others [12], [33].

**Table 2:** Estimated parameters of gene regulatory networks in Table 1 with sixteen genes in the thymus (A) and spinal cord (B) at the young and aged stages.

	Parameters of Young Stage				Parameters of Aged Stage				
**(A) Thymus**	α_1,1_ = 0.407	α_1,4_ = 0.183	α_1,6_ = −0.213	α_1,10_ = −0.031	α_1,1_ = 0.258	α_1,4_ = 0.143	α_1,6_ = −0.023	α_1,10_ = −0.089	
	α_1,11_ = 0.060	α_1,12_ = −0.408	α_2,1_ = 0.099	α_2,2_ = 0.964	α_1,11_ = 0.033	α_1,12_ = −0.401	α_2,1_ = 0.186	α_2,2_ = 0.525	
	α_2,4_ = −0.130	α_2,9_ = 0.036	α_2,13_ = 0.126	α_3,3_ = 0.990	α_2,4_ = 0.408	α_2,9_ = 0.182	α_2,13_ = −0.122	α_3,3_ = 1.002	
	α_3,4_ = −0.071	α_3,6_ = −0.027	α_3,16_ = −0.013	α_4,4_ = 1.050	α_3,4_ = 0.055	α_3,6_ = 0.109	α_3,16_ = 0.100	α_4,4_ = 0.978	
	α_4,5_ = 0.026	α_4,15_ = 0.004	α_5,3_ = −0.001	α_5,5_ = 0.998	α_4,5_ = 0.036	α_4,15_ = 0.04	α_5,3_ = 0.019	α_5,5_ = 0.978	
	α_6,2_ = 0.413	α_6,3_ = 0.125	α_6,6_ = 0.424	α_6,7_ = 0.120	α_6,2_ = −0.087	α_6,3_ = −0.008	α_6,6_ = 0.199	α_6,7_ = −0.174	
	α_6,10_ = 0.200	α_6,16_ = 0.065	α_7,3_ = 0.028	α_7,7_ = 0.937	α_6,10_ = 0.070	α_6,16_ = −0.292	α_7,3_ = −0.093	α_7,7_ = 1.096	
	α_7,15_ = −0.006	α_8,3_ = 0.008	α_8,8_ = 1.106	α_9,8_ = −0.011	α_7,15_ = −0.107	α_8,3_ = 0.05	α_8,8_ = 1.001	α_9,8_ = −0.027	
	α_9,9_ = 0.985	α_9,11_ = −0.001	α_10,9_ = 0.009	α_10,10_ = 1.068	α_9,9_ = 0.982	α_9,11_ = 0.014	α_10,9_ = −0.02	α_10,10_ = 0.998	
	α_10,11_ = −0.024	α_11,2_ = 0.021	α_11,11_ = 1.015	α_12,9_ = 0.012	α_10,11_ = 0.007	α_11,2_ = 0.003	α_11,11_ = 0.994	α_12,9_ = −0.003	
	α_12,12_ = 1.019	α_13,12_ = 0.009	α_13,13_ = 0.998	α_14,3_ = −0.003	α_12,12_ = 1.028	α_13,12_ = 0.029	α_13,13_ = 1.009	α_14,3_ = −0.092	
	α_14,14_ = 1.030	α_15,7_ = 0.176	α_15,14_ = −0.0799	α_15,15_ = 0.964	α_14,14_ = 0.965	α_15,7_ = 0.366	α_15,14_ = 0.253	α_15,15_ = 0.454	
	α_15,16_ = −0.061	α_16,15_ = −0.010	α_16,16_ = 1.014		α_15,16_ = −0.045	α_16,15_ = 0.025	α_16,16_ = 0.997		
**(B) Spinal Cord**	α_1,1_ = 0.388	α_1,4_ = 0.082	α_1,6_ = 0.004	α_1,10_ = −0.161	α_1,1_ = 0.886	α_1,4_ = 0.044	α_1,6_ = 0.085	α_1,10_ = 0.043	
	α_1,11_ = 0.001	α_1,12_ = −0.462	α_2,1_ = −0.022	α_2,2_ = 0.920	α_1,11_ = −0.159	α_1,12_ = −0.101	α_2,1_ = 0.186	α_2,2_ = 0.835	
	α_2,4_ = −0.013	α_2,9_ = 0.120	α_2,13_ = −0.267	α_3,3_ = 0.954	α_2,4_ = 0.069	α_2,9_ = 0.034	α_2,13_ = 0.233	α_3,3_ = 0.869	
	α_3,4_ = 0.005	α_3,6_ = 0.268	α_3,16_ = 0.051	α_4,4_ = 0.979	α_3,4_ = −0.004	α_3,6_ = 0.003	α_3,16_ = −0.195	α_4,4_ = 0.911	
	α_4,5_ = −0.043	α_4,15_ = 0.129	α_5,3_ = 0.080	α_5,5_ = 0.974	α_4,5_ = 0.242	α_4,15_ = −0.364	α_5,3_ = −0.044	α_5,5_ = 0.998	
	α_6,2_ = −0.074	α_6,3_ = 0.084	α_6,6_ = 0.049	α_6,7_ = -.0031	α_6,2_ = 0.140	α_6,3_ = −0.013	α_6,6_ = 0.825	α_6,7_ = −0.289	
	α_6,10_ = −0.152	α_6,16_ = −0.102	α_7,3_ = 0.187	α_7,7_ = 1.040	α_6,10_ = 0.137	α_6,16_ = 0.045	α_7,3_ = 0.003	α_7,7_ = 0.991	
	α_7,15_ = −0.157	α_8,3_ = 0.037	α_8,8_ = 1.009	α_9,8_ = −0.006	α_7,15_ = −0.007	α_8,3_ = −0.019	α_8,8_ = 0.998	α_9,8_ = −0.091	
	α_9,9_ = 1.000	α_9,11_ = −0.008	α_10,9_ = 0.009	α_10,10_ = 0.981	α_9,9_ = 0.816	α_9,11_ = 0.126	α_10,9_ = 0.009	α_10,10_ = 0.980	
	α_10,11_ = 0.034	α_11,2_ = 0.013	α_11,11_ = 1.033	α_12,9_ = 0.018	α_10,11_ = 0.001	α_11,2_ = 0.004	α_11,11_ = 0.992	α_12,9_ = −0.057	
	α_12,12_ = 1.023	α_13,12_ = −0.002	α_13,13_ = 1.015	α_14,3_ = 0.596	α_12,12_ = 0.972	α_13,12_ = 0.007	α_13,13_ = 1.011	α_14,3_ = −0.011	
	α_14,14_ = 0.878	α_15,7_ = −0.064	α_15,14_ = 0.148	α_15,15_ = 0.681	α_14,14_ = 1.002	α_15,7_ = −0.352	α_15,14_ = 0.130	α_15,15_ = 0.667	
	α_15,16_ = 0.256	α_16,15_ = −0.021	α_16,16_ = 1.009		α_15,16_ = −0.205	α_16,15_ = −0.030	α_16,16_ = 1.009		

#### Estimating network robustness and response ability of GRNs at different stages of life by NRM and RAM methods

It has previously been shown that gene networks associated with aging-related pathophysiological phenotypes suffers from intrinsic perturbations mainly due to noise processes like molecular fluctuation or genetic mutation. The robustness of the GRN is defined as its ability to tolerate intrinsic perturbations and plays a principal role as a fail-safe mechanism during evolutionary processes [12]. Therefore, comparisons of robustness 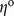 for different GRNs pertaining to young and aged stages may quantitatively describe their relative ability to tolerate these intrinsic perturbations. By using our NRM method, we evaluate the network robustness as shown in Table 3 to represent the maximum intensity of intrinsic perturbations that the network system can tolerate. In other words, the higher the 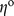 calculated from a

**Table 3:** Estimated network robustness (*η* ^o^) and response ability (*δ* ^o^).

(A)		Young	Aged
**Thymus**	***η*** **^o^**	0.2233	0.3852
	***δ*** **^o^**	1.1770	0.9362
**Spinal Cord**	***η*** **^o^**	0.2360	0.6417
	***δ*** **^o^**	1.1936	0.9073
**(B)**		**Fibroblast**	**HeLa**
**Menadione**	***η*** **^o^**	0.1250	1.0016
**Treatment**	***δ*** **^o^**	1.1524	0.6159

Network robustness (*η* ^o^) and response ability (*δ* ^o^) of the GRN with sixteen genes across different tissues at different stages of life are shown in (A). In addition, the network robustness (*η* ^o^) and response ability (*δ* ^o^) of PPINs evaluated in normal fibroblast and HeLa cancer cells under menadione treatment are indicated in (B).

Concerning the RAM, if a GRN also responds to external stimuli 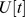, such as upstream signals or biological molecules outside the network, as in equation (11), the network response ability measured should be based on the output/input energy ratio within a time interval as detailed in equation (12). Supposing the ratio of the effect of input signals 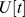 on 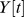 is upper-bounded by a positive value 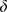
_,_ then we can calculate the smallest value 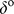 in equation (12) (i.e., the minimum upper bound), and call it the “network response ability” to external stimuli. This method, RAM, is based on system gain theory, and incorporates principles behind the external input signal in system theory perspectives [27]. In general, a GRN with greater robustness always has lower response ability, and vice versa. This is an inherent characteristic of physical systems [27]. The trade-off between robustness and response ability is demonstrably visible in Table 3 and constitutes secondary proof of our proposed method.

Comparing the GRNs of the thymus and spinal cord data between the “young” group and the “aged” group, we show that both tissues evaluated have a higher robustness 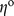 value in the aged group than the young group. These results are shown in Table 3. The aging-related GRN examined in the first case study is less robust to intrinsic perturbations in the young group than in the aged group. In other words, the GRN at the aged stage can function in spite of the accumulation of DNA damage or genetic mutations accompanying the aging process. In addition, response ability (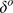) of the GRN calculated from the aged group is lower than that from the young group (Table 3A). In order to tolerate and survive the accumulated intrinsic perturbations (e.g., genetic mutations) associated with the increase in age, the elderly GRN becomes more robust than the young GRN. Therefore, elderly GRNs are less able to respond to external stimuli or transduce them downstream. Hence, the response ability protecting organismal function against external stimuli (e.g., pro-inflammatory molecules) may become worse in the elderly GRN. To confirm the power of the proposed methods, the proposed network robustness and response ability is validated by computer simulation in Figure S1–S3 in Text S7.

In addition, a more robust GRN could harbor more accumulated genetic mutations, which might provide more evolutionary paths to other GRN phenotypes via random drift, and even lead to aging-related diseases like cancers, metabolic disorders, and arthritis [34], [35]. On the other hand, the young aging-related GRN is less robust, with a greater response to external stimuli. From the above data, we can conclude that the GRNs of the young group more efficiently respond to external stimuli. These values show that while the GRN of the young group is less robust to intrinsic perturbations, external stimuli elicit a strong response from it, suggesting that gene expression may be readily reprogrammed to mediate downstream genes or regulators. This also allows for modulation of gene expression in response to external stimuli such as exposure to oxidative stress, carcinogens, and pro-inflammatory molecules.

### Case 2: Estimating the Robustness and Response Ability of a Protein-protein Interaction Network (PPIN) between Normal and Cancer Cells

Robustness, an intrinsic and systematic property of complex biological systems, enables living organisms to maintain their functions in the face of various perturbations to assure their survival [4], [12], [36]. Moreover, most genes code for sensors, actuators, and the complex regulatory networks that control them, and thus confer robustness to the cell under various challenging circumstances rather than providing merely the basic functionality required for survival under a single, ideal environment. Normal as well as cancer cells utilize the same processes. At present, although researchers have contended that cancer is a highly robust disease, and clinical observations suggest that inefficiencies in cancer therapy may be due to the according drug resistance, a useful computational method to evaluate the network robustness from microarray data and compare it between cancer and normal cells remains lacking. Therefore, in the second case, the NRM method we have proposed is applied to calculate the PPIN robustness of HeLa cells versus normal fibroblasts under menadione treatment (i.e., exposed to oxidative stress) from corresponding microarray data and protein interaction information. The network response ability is also evaluated and discussed.

#### Constructing refined PPINs of normal fibroblast cells and HeLa cancer cells under menadione treatment

We use microarray data downloaded from the Gene Expression Omnibus (GEO) database on the NCBI website in this study to investigate the network robustness and response ability of a PPIN in normal cells versus cancer cells under oxidative stress [37]. Normal human diploid lung fibroblasts and HeLa S3 cervical carcinoma cells are treated with 10 µM menadione bisulfate for 0.5, 1, 3, 4, 6, 8, 12, 24, and 36 hours and 0.5, 1, 2, 4, 8, 12, 24, and 32 hours, respectively. For the oxidative stress condition, the temporal responses at several time points over an interval of 24–36 hours are measured, allowing stress effects to be observed over an interval longer than a normal cell cycle duration.

**Figure 4:**
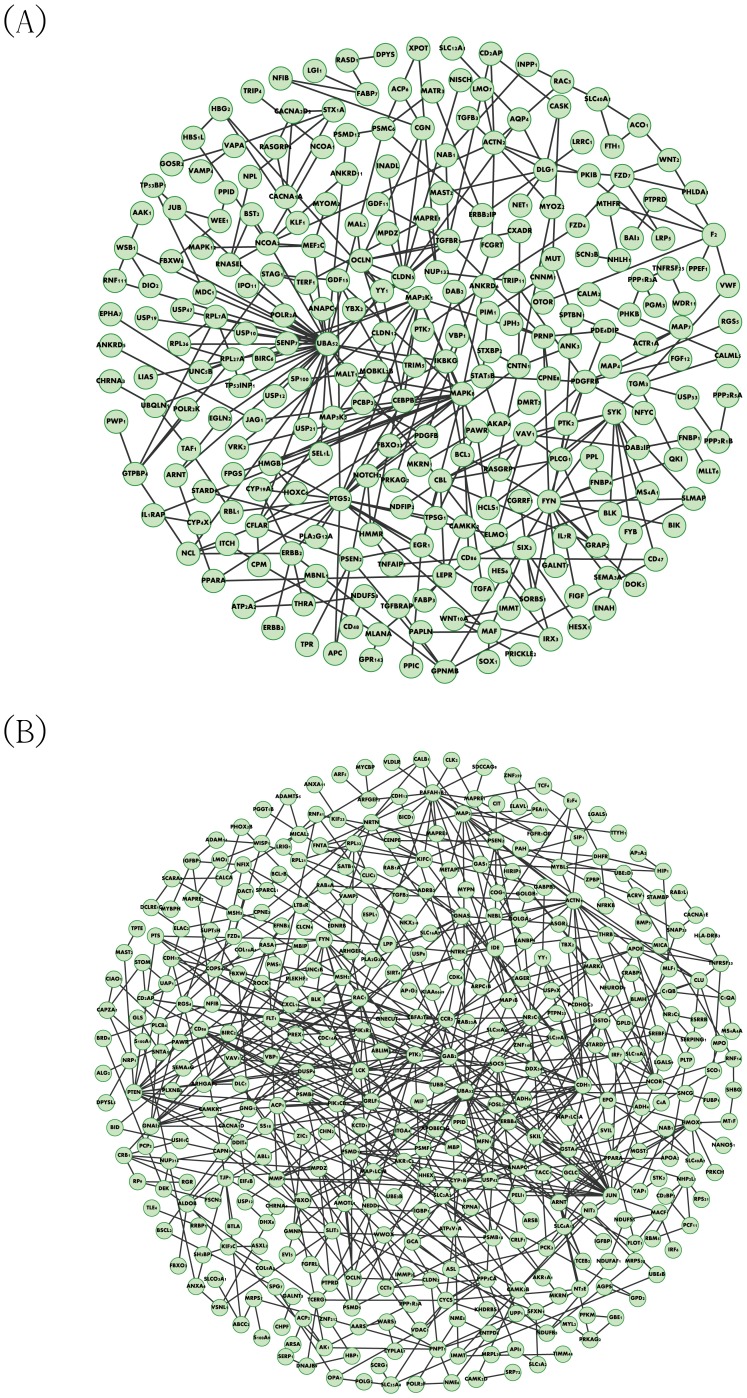
Refined protein-protein interaction network. The figure shows the refined PPIN with effective protein interactions under oxidative stress in fibroblast (A) and HeLa cells (B). A dynamic PPI model and model selection method, Akaike Information Criterion (AIC), are used together to prune the rough PPIN using the time series microarray data to delete unrealistic and false positive PPIs in the PPIN.

As shown in the flowchart in Figure 1, the microarray data are integrated with database information concerning protein interactions as the input for our proposed method (see Materials & Methods). The candidate protein interactions are taken from all experimental conditions in these data to construct the rough PPIN. To proceed further, the corresponding microarray data are used to identify the parameters in equation (8) via the recursive least square estimation method [17], and then to prune down the rough PPINs based on these identified parameters, thus obtaining the refined PPINs of both fibroblast and HeLa cells under menadione treatment via the AIC (shown in Figure 4) [22]. After obtaining the refined PPINs, we can see there are still 161 candidate proteins with 371 interactions in the fibroblast PPIN and 222 candidate proteins with 607 interactions in the HeLa PPIN under oxidative stress conditions. Furthermore, the respective system interactive matrices *R* of the fibroblast and HeLa cell PPINs can be acquired and then applied to the sequential evaluation of network robustness by equation (10) and response ability by equation (15).

#### Different network robustness and response ability between the PPINs of normal fibroblasts and HeLa cells both under oxidative stress

We compare the PPINs of fibroblasts and HeLa cells while both are under an oxidative stress insult and show that the PPIN of HeLa cells possesses a higher robustness 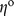 value than the PPIN of fibroblasts using our NRM method. These results are shown in Table 3B. The PPIN is more robust to such perturbation in the cancer cells than in the normal cells. Moreover, the response ability (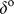) of the PPIN calculated from the HeLa cells is lower than that from the fibroblasts (Table 3B). Therefore, the PPIN of cancer cells is less efficient in transducing or responding to external stimuli when compared with that of normal cells. The analytic results support previous contentions that cancer cells are more robust than normal cells [38].

Accordingly, compelling evidence has previously suggested that the level of reactive oxygen species (ROS) is higher in many types of cancer cells than in their normal counterparts [39], [40], [41]. Although a moderate increase in ROS can promote cell proliferation and differentiation, excessive amounts can cause oxidative damage to DNA, protein, and lipids. However, during tumorigenesis or advanced malignant transformation, oncogenic signals can elevate cellular levels of ROS to the extent that numerous genes are altered, accumulating changes via both genetic and epigenetic mechanisms [42], [43]. Therefore, cancer cells themselves actively generate high levels of endogenous ROS and are persistently exposed to them, stimulating cell proliferation through redox-sensitive transcription factors and promoting anti-oxidant adaptive mechanisms to minimize oxidative damage [44], [45]. Therefore, the intrinsic oxidative stress generated during tumor initiation or malignant transformation may exert selective pressure on the cell population and enrich those capable of stress adaptation: i.e., cancer cells. These cells that survive oxidative stress may have adapted mechanisms to counteract the cytotoxic effects of increased ROS and to promote pathways essential to cellular survival [45]. All of these alterations confer cellular resistance and system robustness to subsequent intrinsic or extrinsic oxidative stress, even at high levels. In clinical cancer therapy, one chemotherapeutic strategy is to elevate excess cellular oxidative stress in order to deplete the antioxidant system and overwhelm the cancer system’s ability to neutralize and eliminate such insults, inducing apoptosis and destroying the cancer network system. However, the efficiency of such chemo- or radiotherapy is still unsatisfactory. Moreover, previous evidence has suggested that tumors are highly robust and maintain their proliferative potential against a wide range of anti-cancer therapies. Heterogeneities have been regarded as a vital property of cancer cells because they facilitate robustness through redundancy, and so subsystems killed by chemotherapy can be functionally replaced to ensure tumor proliferation and survival [38]. Hence, the notion of the PPIN of HeLa cells having higher robustness, as evaluated by our proposed NRM method, is consistent with previous contentions and experimental observations. Utilizing microarray data in system-level computations, we have sufficiently demonstrated that cancer cells possess better robustness and less response ability under oxidative stress when compared with normal cells.

### Conclusions

Our proposed methods, NRM and RAM, consist of multiple analytical steps, from constructing dynamic GRNs and PPINs via microarray data to estimating network robustness and response ability based on their dynamic network systems. However, each individual step can be employed separately, depending on the specific application in question. For example, in the first step we select a set of differentially expressed genes in aging (Case 1) or cancer (Case 2) as our target genes. However, another user could replace this set of genes with other sets of genes of interest to facilitate targeted studies over a wide range of issues. In addition, our proposed methods can evaluate the network robustness and response ability not only for GRNs (Case 1) but also for PPINs (Case 2) depending on the needs of the user. The generalized NRM and RAM methods are effective tools for exploring and analyzing different systems-level dynamical properties. Making use of such network properties can facilitate biomedical predictions and applications, providing useful information for clinical strategy, drug target selection, and design specifications of synthetic biology from the perspective of systems biology [10], [14], [15], [16].

## Supporting Information

Text S1 Parameter Identification of a Gene Regulatory Network by Microarray Data.
(DOC)

Text S2 Network Parameter Identification of PPIN and Pruning of False Positive Protein Interactions.
(DOC)

Text S3 Proof of Proposition 1.
(DOC)

Text S4 Proof of Proposition 2.
(DOC)

Text S5 Proof of Proposition 3.
(DOC)

Text S6 Proof of Proposition 4.
(DOC)

Text S7 Computer Simulation of the Proposed Network Robustness and Response Ability Methods.
(DOC)

## References

[1] Mandel EM, Grosschedl R (2010) Transcription control of early B cell differentiation. Curr Opin Immunol 22: 161–167. 20144854 10.1016/j.coi.2010.01.010

[2] Storey JD, Xiao W, Leek JT, Tompkins RG, Davis RW (2005) Significance analysis of time course microarray experiments. Proc Natl Acad Sci U S A 102: 12837–12842. 16141318 10.1073/pnas.0504609102 PMC1201697

[3] Zhu D, Hero AO, Cheng H, Khanna R, Swaroop A (2005) Network constrained clustering for gene microarray data. Bioinformatics 21: 4014–4020. 16141248 10.1093/bioinformatics/bti655

[4] Stelling J, Sauer U, Szallasi Z, Doyle FJ 3rd, Doyle J (2004) Robustness of cellular functions. Cell 118: 675–685. 15369668 10.1016/j.cell.2004.09.008

[5] Chen BS, Lee TS, Feng JH (1994) A Nonlinear H∞ control design in robotic systems under parameter perturbation and external disturbance. International Journal of Control 59: 439–461.

[6] Hopfield JJ (1974) Kinetic proofreading: a new mechanism for reducing errors in biosynthetic processes requiring high specificity. Proc Natl Acad Sci U S A 71: 4135–4139. 4530290 10.1073/pnas.71.10.4135 PMC434344

[7] Yan J, Magnasco MO, Marko JF (2001) Kinetic proofreading can explain the supression of supercoiling of circular DNA molecules by type-II topoisomerases. Phys Rev E Stat Nonlin Soft Matter Phys 63: 031909. 11308680 10.1103/PhysRevE.63.031909

[8] Pastor-Satorras R, Vespignani A (2004) Evolution and structure of the Internet: Cambridge University Press.

[9] Angeli D, Ferrell JE Jr, Sontag ED (2004) Detection of multistability, bifurcations, and hysteresis in a large class of biological positive-feedback systems. Proc Natl Acad Sci U S A 101: 1822–1827. 14766974 10.1073/pnas.0308265100 PMC357011

[10] Hasty J, McMillen D, Collins JJ (2002) Engineered gene circuits. Nature 420: 224–230. 12432407 10.1038/nature01257

[11] Zhang S, Jin G, Zhang XS, Chen L (2007) Discovering functions and revealing mechanisms at molecular level from biological networks. Proteomics 7: 2856–2869. 17703505 10.1002/pmic.200700095

[12] Kitano H (2004) Biological robustness. Nat Rev Genet 5: 826–837. 15520792 10.1038/nrg1471

[13] Doyle JC Francis B, Tannenbaum A (1992) Feedback Control Theory: Macmillan Publishing Company.

[14] Kaern M, Blake WJ, Collins JJ (2003) The engineering of gene regulatory networks. Annu Rev Biomed Eng 5: 179–206. 14527313 10.1146/annurev.bioeng.5.040202.121553

[15] Murphy KF, Balazsi G, Collins JJ (2007) Combinatorial promoter design for engineering noisy gene expression. Proc Natl Acad Sci U S A 104: 12726–12731. 17652177 10.1073/pnas.0608451104 PMC1931564

[16] Chen BS, Wu CH (2009) A systematic design method for robust synthetic biology to satisfy design specifications. BMC Syst Biol 3: 66. 19566953 10.1186/1752-0509-3-66 PMC2732592

[17] Johansson R (1993) System modeling and identification.

[18] Noble WS (2009) How does multiple testing correction work? Nature Biotechnology 27: 1135–1137. 10.1038/nbt1209-1135 PMC2907892 20010596

[19] Bland JM, Altman DG (1995) Multiple Significance Tests - the Bonferroni Method.10. British Medical Journal 310: 170–170. 7833759 10.1136/bmj.310.6973.170 PMC2548561

[20] Gandhi TK, Zhong J, Mathivanan S, Karthick L, Chandrika KN, et al (2006) Analysis of the human protein interactome and comparison with yeast, worm and fly interaction datasets. Nat Genet 38: 285–293. 16501559 10.1038/ng1747

[21] Troyanskaya OG (2005) Putting microarrays in a context: integrated analysis of diverse biological data. Brief Bioinform 6: 34–43. 15826355 10.1093/bib/6.1.34

[22] Akaike H (1974) A new look at the statistical model identification. Automatic Control, IEEE Transactions on 19: 716–723.

[23] Klipp E KA, Wierling C, Lehrach H (2005) Systems Biology in Practice. Concepts, Implementation and Application. Berlin: Wiley-VCH.

[24] Kuo BC, Golnaraghi F (2009) Automatic Control Systems: John Wiley & Sons Inc.

[25] Chen BS, Zhang W (2004) Stochastic H2/H∞ control with state-dependent noise. IEEE Trans Automatic Control 49: 45–57.

[26] Zhang W, Chen BS (2006) State feedback H∞ control for a class of nonlinear stochastic systems. SIAM J on Control and Optimization 44: 1973–1991.

[27] Boyd SP (1994) Linear matrix inequalities in system and control theory. Philadelphia: Society for Industrial and Applied Mathematics. ix, 193 p. p.

[28] Lin CL, Lai CC, Huang TH (2000) A neural network for linear matrix inequality problems. IEEE Trans Neural Netw 11: 1078–1092. 18249836 10.1109/72.870041

[29] Chung HY, Sung B, Jung KJ, Zou Y, Yu BP (2006) The molecular inflammatory process in aging. Antioxid Redox Signal 8: 572–581. 16677101 10.1089/ars.2006.8.572

[30] Judge S, Jang YM, Smith A, Hagen T, Leeuwenburgh C (2005) Age-associated increases in oxidative stress and antioxidant enzyme activities in cardiac interfibrillar mitochondria: implications for the mitochondrial theory of aging. FASEB J 19: 419–421. 15642720 10.1096/fj.04-2622fje

[31] Zahn JM, Poosala S, Owen AB, Ingram DK, Lustig A, et al (2007) AGEMAP: a gene expression database for aging in mice. PLoS Genet 3: e201. 18081424 10.1371/journal.pgen.0030201 PMC2098796

[32] Cheadle C, Vawter MP, Freed WJ, Becker KG (2003) Analysis of microarray data using Z score transformation. J Mol Diagn 5: 73–81. 12707371 10.1016/S1525-1578(10)60455-2 PMC1907322

[33] Tegner J, Yeung MK, Hasty J, Collins JJ (2003) Reverse engineering gene networks: integrating genetic perturbations with dynamical modeling. Proc Natl Acad Sci U S A 100: 5944–5949. 12730377 10.1073/pnas.0933416100 PMC156306

[34] Lenski RE, Barrick JE, Ofria C (2006) Balancing robustness and evolvability. PLoS Biol 4: e428. 17238277 10.1371/journal.pbio.0040428 PMC1750925

[35] de Visser JA, Hermisson J, Wagner GP, Ancel Meyers L, Bagheri-Chaichian H, et al (2003) Perspective: Evolution and detection of genetic robustness. Evolution 57: 1959–1972. 14575319 10.1111/j.0014-3820.2003.tb00377.x

[36] Csete ME, Doyle JC (2002) Reverse engineering of biological complexity. Science 295: 1664–1669. 11872830 10.1126/science.1069981

[37] Murray JI, Whitfield ML, Trinklein ND, Myers RM, Brown PO, et al (2004) Diverse and specific gene expression responses to stresses in cultured human cells. Mol Biol Cell 15: 2361–2374. 15004229 10.1091/mbc.E03-11-0799 PMC404029

[38] Kitano H (2004) Cancer as a robust system: implications for anticancer therapy. Nat Rev Cancer 4: 227–235. 14993904 10.1038/nrc1300

[39] Toyokuni S, Okamoto K, Yodoi J, Hiai H (1995) Persistent oxidative stress in cancer. FEBS Lett 358: 1–3. 7821417 10.1016/0014-5793(94)01368-b

[40] Kawanishi S, Hiraku Y, Pinlaor S, Ma N (2006) Oxidative and nitrative DNA damage in animals and patients with inflammatory diseases in relation to inflammation-related carcinogenesis. Biol Chem 387: 365–372. 16606333 10.1515/BC.2006.049

[41] Szatrowski TP, Nathan CF (1991) Production of large amounts of hydrogen peroxide by human tumor cells. Cancer Res 51: 794–798. 1846317

[42] Schneider BL, Kulesz-Martin M (2004) Destructive cycles: the role of genomic instability and adaptation in carcinogenesis. Carcinogenesis 25: 2033–2044. 15180945 10.1093/carcin/bgh204

[43] Wallace DC (2005) The mitochondrial genome in human adaptive radiation and disease: on the road to therapeutics and performance enhancement. Gene 354: 169–180. 16024186 10.1016/j.gene.2005.05.001

[44] Irmak MB, Ince G, Ozturk M, Cetin-Atalay R (2003) Acquired tolerance of hepatocellular carcinoma cells to selenium deficiency: a selective survival mechanism? Cancer Res 63: 6707–6715. 14583465

[45] Trachootham D, Alexandre J, Huang P (2009) Targeting cancer cells by ROS-mediated mechanisms: a radical therapeutic approach? Nat Rev Drug Discov 8: 579–591. 19478820 10.1038/nrd2803

