# Supplementary material for: New Measurement Methods of Network Robustness and Response Ability via Microarray Data
Source: PLoS One. 2013 Jan 28;8(1):e55230. doi: 10.1371/journal.pone.0055230 (PMC3557243; doi:10.1371/journal.pone.0055230)
Supplement: Text S1 — Parameter Identification of a Gene Regulatory Network by Microarray Data. (DOC) [file pone.0055230.s001.doc]

**Text S1. Parameter Identification of a Gene Regulatory Network by Microarray Data**

The focus of our investigation is whether the robustness and response ability of a GRN would be distinct and dependent on dynamic behavior under different biological conditions or stages. For this reason, the application of our proposed measurement method for robustness and response ability is highly dependent on the GRN dynamic model, which can be constructed from the time profiles of microarray data at these different stages. In general, for the parameter estimation method for the GRN dynamic model, the amount of microarray data for parameter estimation must be much (i.e., at least 3 times) larger than the number of parameters to be estimated , or it will lead to overfitting due to the data regression vector becoming singular in the parameter estimation process. That is, the overfitting due to the shortage of data points will lead to a large distortion in the parameter estimation of the GRN dynamic model. Therefore, we use the recursive least square estimation method to estimate the interactive matrix $A$ and basal level $H$ in (1) from the time-series microarray data. Based on the least square parameter estimation in , let us denote $V(\hat{\theta})=\sum_{t=1}^{M} \left(X(t+1)-\hat{A}X(t)-\hat{H}\right)^{T}\left(X(t+1)-\hat{A}X(t)-\hat{H}\right)=\sum_{t=1}^{M} \varepsilon^{T}(t)\varepsilon(t)$ where $\hat{\theta}=\left[\begin{matrix} \hat{A} \\ \hat{H} \end{matrix}\right]$ denotes the estimate of parameters $\theta=\left[\begin{matrix} A \\ H \end{matrix}\right]$, and *M* is the amount of microarray data for parameter estimation. Using the recursive least square parameter estimation algorithm, we can estimate $\hat{\theta}$ from the microarray data. In order to improve the parameter estimate when only small amounts of microarray data are available, the recursive least square algorithm is performed iteratively for several rounds, with the estimated parameters of each round as the initial values of the next round, repeated until the estimated parameters converge. After the recursive least square parameter estimation of the final round, the covariance of estimation error $\varepsilon(t)$ is given by $Cov(\varepsilon)=\frac{2}{M-2}V(\hat{\theta})$ and the covariance matrix of $\hat{\theta}$ is given by $Cov(\hat{\theta})=Cov(\varepsilon)\left[\sum_{t=1}^{M} X^{T}(t)X(t)+I\right]^{-1}.$These parameter estimation methods for constructing GRNs are termed "reverse engineering methods" in systems biology . Based on the dynamic model described above and on microarray data, the system parameters *A* for GRNs can be estimated for use in NRM and RAM methods.
