# Supplementary material for: New Measurement Methods of Network Robustness and Response Ability via Microarray Data
Source: PLoS One. 2013 Jan 28;8(1):e55230. doi: 10.1371/journal.pone.0055230 (PMC3557243; doi:10.1371/journal.pone.0055230)
Supplement: Text S2 — Network Parameter Identification of PPIN and Pruning of False Positive Protein Interactions. (DOC) [file pone.0055230.s002.doc]

**Text S2. Network Parameter Identification of PPIN and Pruning of False Positive Protein Interactions**

After constructing the discrete dynamic model (2) for all possible proteins in the rough PPIN, we use the least square estimation method to estimate the interaction parameters *a_ii_*, *b_ij_*, and *k_i_* in (2) from time-series microarray data. Hence, we can rewrite equation (2) in the following regression form:

where *Q* denotes the number of possible interactions with the target protein and stands for the regression vector obtained from time series microarray data in the above process. is the parameter vector of the PPIN to be estimated.

By computing equation (A1) at different time points, we can construct the following vector-form equation:

where *M* denotes the number of microarray data points. These data points are utilized as the basis of our regression vector. For simplicity, we further represent the above equation as follows:

We then use the recursive least square estimation method to estimate using regression data acquired from microarray data of the *i*th target protein and its corresponding interacting proteins.

After obtaining the estimated parameter by the recursive least square estimation method, the estimated PPI in equation (2) can be written as follows:

In equation (A4), the parameter represents the estimation of the transition rate of the target protein, and denotes the individual interactive rate between protein *j* and the target protein *i*. A positive value implies positive interaction, while a negative value implies negative interaction, and both interactions become more likely as the parameters get larger.

After identifying the parameters of the dynamic model in equation (2) through several rounds of the recursive least square estimation method, the reality of the interaction is still unknown, as is whether it can be considered true. Therefore, in order to determine whether a PPI (i.e., in (A4)) is realistic or simply a false positive in the rough PPIN, we propose a statistical approach based on model order detection for evaluating the reality of interaction parameters in the rough PPIN and pruning from it the false positive PPIs. Here, we employ the Akaike Information Criterion (AIC) to validate the model order, or the number of PPI parameters of the network. The AIC, a method for model order selection, attempts to include both the estimated residual variance and model complexity in one statistic. AIC decreases as residual variance decreases, and increases as the number of parameters increases. As the expected residual variance decreases with increasing parameter numbers for excessive model complexity, a minimum should appear near the correct parameter number . For a protein interaction dynamic model with *Q* interaction parameters to fit with data from *N* samples, the AIC can be written as follows:

where denotes the estimated expression profile of the *i*th target protein: i.e., . After the statistical selection of *Q* interaction parameters derived from minimizing the AIC, we can determine whether the PPI is realistic or false positive for target protein *i*. After a number test of interaction parameters in by using the AIC system order test for all target proteins, we can construct a realistic dynamic PPIN in (3) from microarray data.
