# Supplementary material for: New Measurement Methods of Network Robustness and Response Ability via Microarray Data
Source: PLoS One. 2013 Jan 28;8(1):e55230. doi: 10.1371/journal.pone.0055230 (PMC3557243; doi:10.1371/journal.pone.0055230)
Supplement: Text S3 — Proof of Proposition 1. (DOC) [file pone.0055230.s003.doc]

**Text S3. Proof of Proposition 1**

Let us denote the Lyapunov (energy) function of perturbative gene regulatory network in equation (8) as $V\left(\tilde{X}\left[t\right]\right)=\tilde{X}^{T}\left[t\right]P\tilde{X}\left[t\right]$, for some symmetric positive definite matrix $P=P^{T}>0$.

$$\begin{matrix} \Delta V(\tilde{X}\left[t\right]) \\ =V\left(\tilde{X}\left[t+1\right]\right)-V(\tilde{X}\left[t\right]) \\ =\tilde{X}^{T}\left[t+1\right]P\tilde{X}\left[t+1\right]-\tilde{X}^{T}\left[t\right]P\tilde{X}\left[t\right] \\ =\tilde{X}^{T}\left[t\right]\left[A(1+\eta)\right]^{T}P\left[A(1+\eta)\right]\tilde{X}\left[t\right]-\tilde{X}^{T}\left[t\right]P\tilde{X}\left[t\right] \\ =\tilde{X}^{T}\left[t\right]\left\{\text{ }\left[A(1+\eta)\right]^{T}P\left[A(1+\eta)\right]-P\text{ }\right\}\tilde{X}\left[t\right] \\ \text{If}\left[A(1+\eta)\right]^{T}P\left[A(1+\eta)\right]-P\leq0,\text{ then }\Delta V(\tilde{X}\left[t\right])\leq0 \end{matrix}$$

Based on quadratic stability , if the LMI in (9) holds, then the perturbative network is robustly stable.
