# Supplementary material for: New Measurement Methods of Network Robustness and Response Ability via Microarray Data
Source: PLoS One. 2013 Jan 28;8(1):e55230. doi: 10.1371/journal.pone.0055230 (PMC3557243; doi:10.1371/journal.pone.0055230)
Supplement: Text S5 — Proof of Proposition 3. (DOC) [file pone.0055230.s005.doc]

**Text S5. Proof of Proposition 3**

Following the proof in Text S3, let us choose Lyapunov function as $V\left(\tilde{X}\left[t\right]\right)=\tilde{X}^{T}\left[t\right]P\tilde{X}\left[t\right]$ for some symmetric positive definite matrix $P=P^{T}>0$. By the fact (21) and (22), we get

Based on quadratic stability , if the LMI in (23) holds, then the perturbative nonlinear network system is robustly stable.
