# Supplementary material for: New Measurement Methods of Network Robustness and Response Ability via Microarray Data
Source: PLoS One. 2013 Jan 28;8(1):e55230. doi: 10.1371/journal.pone.0055230 (PMC3557243; doi:10.1371/journal.pone.0055230)
Supplement: Text S6 — Proof of Proposition 4. (DOC) [file pone.0055230.s006.doc]

**Text S6. Proof of Proposition 4**

Following the proof in Text S4, we choose Lyapunov $V\left(\tilde{X}\left[t\right]\right)=\tilde{X}^{T}\left[t\right]P\tilde{X}\left[t\right]$, for some symmetric positive definite matrix $P=P^{T}>0$. We have

If the following inequality holds:

$\begin{matrix} \tilde{X}^{T}\left[t\right]A_{i}^{T}PA_{i}\tilde{X}\left[t\right]+\tilde{X}^{T}\left[t\right]A_{i}^{T}PU\left[t\right]+U^{T}\left[t\right]PA_{i}\tilde{X}\left[t\right]+U^{T}\left[t\right]PU\left[t\right] \\ +\tilde{X}^{T}\left[t\right]C^{T}C\tilde{X}\left[t\right]-\delta^{2}U^{T}\left[t\right]U\left[t\right]-\tilde{X}^{T}\left[t\right]P\tilde{X}\left[t\right]\leq0 \end{matrix}$ (A10)

then, we get $V\left(\tilde{X}\left[t+1\right]\right)\leq V\left(\tilde{X}\left[t\right]\right)-Y^{T}[t]Y[t]+\delta^{2}U^{T}\left[t\right]U\left[t\right]$

So we sum the above inequality from 0 to ∞ to get

$$\begin{matrix} V\left(\tilde{X}\left[\infty\right]\right)\leq V\left(\tilde{X}\left[0\right]\right)-\sum_{t=0}^{\infty} Y^{T}[t]Y[t]+\delta^{2}\sum_{t=0}^{\infty} U^{T}\left[t\right]U\left[t\right] \\ \Rightarrow\text{ }V\left(\tilde{X}\left[\infty\right]\right)+\sum_{t=0}^{\infty} Y^{T}[t]Y[t]\leq V\left(\tilde{X}\left[0\right]\right)+\delta^{2}\sum_{t=0}^{\infty} U^{T}\left[t\right]U\left[t\right] \\ \Rightarrow\text{ }\sum_{t=0}^{\infty} Y^{T}[t]Y[t]\leq V\left(\tilde{X}\left[\infty\right]\right)+\sum_{t=0}^{\infty} Y^{T}[t]Y[t]\leq V\left(\tilde{X}\left[0\right]\right)+\delta^{2}\sum_{t=0}^{\infty} U^{T}\left[t\right]U\left[t\right]\text{ (A11)} \end{matrix}$$

$$\begin{matrix} \Rightarrow\text{ }\sum_{t=0}^{\infty} Y^{T}[t]Y[t]\leq\delta^{2}\sum_{t=0}^{\infty} U^{T}\left[t\right]U\left[t\right]\text{ or }\frac{\sum_{t=0}^{\infty} Y^{T}[t]Y[t]}{\sum_{t=0}^{\infty} U^{T}\left[t\right]U\left[t\right]}\leq\delta^{2}\text{ , if }V\left(\tilde{X}\left[0\right]\right)\text{=0 in the } \\ \text{ case }\tilde{X}\left[0\right]\text{=0 } \end{matrix}$$

If the $V\left(\tilde{X}\left[0\right]\right)\neq0\text{ }$in the case $\tilde{X}\left[0\right]\neq0$, then the inequality in (A11) should be modified as

$\sum_{t=0}^{\infty} Y^{T}[t]Y[t]\leq\tilde{X}^{T}\text{[0]}P\tilde{X}[0]+\delta^{2}\sum_{t=0}^{\infty} U^{T}\left[t\right]U\left[t\right]$ (A12)

The above results hold only when the inequality (A10) holds, i.e., the following inequality holds:

$$\begin{matrix} \tilde{X}^{T}\left[t\right]A_{i}^{T}PA_{i}\tilde{X}\left[t\right]+\tilde{X}^{T}\left[t\right]A_{i}^{T}PU\left[t\right]+U^{T}\left[t\right]PA_{i}\tilde{X}\left[t\right]+U^{T}\left[t\right]PU\left[t\right] \\ +\tilde{X}^{T}\left[t\right]C^{T}C\tilde{X}\left[t\right]-\delta^{2}U^{T}\left[t\right]U\left[t\right]-\tilde{X}^{T}\left[t\right]P\tilde{X}\left[t\right]\leq0 \end{matrix}$$

The above inequality is equivalent to the following inequality

$$\left[\begin{matrix} \tilde{X} \\ U \end{matrix}\right]^{T}\left[\begin{matrix} A_{i}^{T}PA_{i}+C^{T}C-P & A_{i}^{T}P \\ PA_{i} & P-\delta^{2}I \end{matrix}\right]\left[\begin{matrix} \tilde{X} \\ U \end{matrix}\right]\leq0\text{ (A13)}$$

The inequality (A13) for all $\tilde{X}\left[t\right]$ and $U\left[t\right]$ if and only if the following LMIs hold

$$\left[\begin{matrix} A_{i}^{T}PA_{i}+C^{T}C-P & A_{i}^{T}P \\ PA_{i} & P-\delta^{2}I \end{matrix}\right]\leq0,\text{ }i=1,\text{ }2,\text{ }\cdots m\text{ }$$
