# Supplementary material for: New Measurement Methods of Network Robustness and Response Ability via Microarray Data
Source: PLoS One. 2013 Jan 28;8(1):e55230. doi: 10.1371/journal.pone.0055230 (PMC3557243; doi:10.1371/journal.pone.0055230)
Supplement: Text S7 — Computer Simulation of the Proposed Network Robustness and Response Ability Methods. (DOC) [file pone.0055230.s007.doc]

In order to confirm the results of proposed approaches, two simulations are given in illustration, one for the confirmation of network robustness and another for the confirmation of network response ability as follows:

Simulation 1:

From the network robustness results of Section 3.1.2 in Table 3, the network robustness of aging-related GRN in thymus tissue is η^o^=0.2233 at the young stage and is η^o^=0.3852 at the aged stage, i.e. the maximum intrinsic perturbations η in (13) could be tolerated by GRNs in young thymus and aged thymus are 0.2233 and 0.3852, respectively. Therefore, according to the proposed network robustness, if an intrinsic perturbation η=0.3, it could not be tolerated by GRN in young thymus, but could be tolerated by GRN in aged thymus. In the simulation results in Figure S1 and Figure S2 in supplementary Text S5, the gene expressions of two GRNs without intrinsic perturbation are shown in Figure S1(A) and Figure S2(A), respectively. However, under the intrinsic perturbation η=0.3, it is seen that the some gene expressions of GRN in young thymus become divergent (i.e. Sirt1, Mdm2, Akt and ATM in Figure S1(B)) while all gene expressions of GRN in aged thymus are still robust in Figure S2(B). These simulation results obviously confirm the proposed network robustness of aging-related GRNs by the proposed method.

Simulation 2:

Similarly, from the network response ability results in Table 3, it is seen that the network response ability of young thymus is larger than that of aged thymus. In order to confirm these results, the response abilities to an unit step signal for sixteen genes of GRNs in young and aged thymus are obtained in Figure S3(A) based on computer simulation, respectively. In general, the gene response abilities of most genes in young thymus are larger than aged thymus. From Figure S3(B), it is seen that the network response ability of GRN in young thymus is larger than that in aged thymus. These simulation results also confirm the proposed network response ability of aging-related GRNs.

Figure S1. Gene expressions of aging-related GRN in thymus at young stage without intrinsic perturbation in (A) and under intrinsic perturbationη=0.3 in (B).

1. Gene expressions of GRN at young stage without intrinsic perturbation

1. Gene expressions of GRN at young stage under intrinsic perturbation

η=0.3>η^o^=0.2233

Figure S2. Gene expressions of aging-related GRN in thymus at aged stage without intrinsic perturbation in (A) and under intrinsic perturbationη=0.3 in (B)

1. Gene expressions of GRN at aged stage without intrinsic perturbation

1. Gene expressions of GRN at aged stage under intrinsic perturbation

η=0.3<η^o^=0.3852

Figure S3. Response ability under external input of unit step signal. (A) Gene response abilities of sixteen genes in GRNs of young and aged thymus, respectively. (B) Network response abilities in GRNs of young and aged thymus, respectively.

(A) Gene response abilities of sixteen genes in GRNs of young and aged thymus, respectively. Obviously, the response abilities of most genes of young thymus are larger than those of aged thymus.

(B) Network response abilities in GRNs of young and aged thymus, respectively. Obviously, the network response ability of young thymus is larger than the aged thymus. The response abilities in this simulation are larger than the estimated response abilities in Table 3 because the estimated response ability δ^o^ in (20) is the lower bound value for all possible input signals. However, the simulated results in this case is only based on the unit step input signal and therefore can not achieve their lower bounds.
